# Recommendations on the follow‐up of patients with Gaucher disease in Spain: Results from a Delphi survey

**DOI:** 10.1002/jmd2.12342

**Published:** 2022-11-08

**Authors:** Pilar Giraldo, Marcio Andrade‐Campos, Montserrat Morales, the SEGA (SEguimiento del paciente de GAucher, Gaucher patient follow‐up) Group

**Affiliations:** ^1^ Hematology Hospital Quironsalud Zaragoza Spain; ^2^ Fundación Española para el Estudio y Tratamiento de la Enfermedad de Gaucher (FEETEG) Zaragoza Spain; ^3^ Hematology Institut Hospital del Mar d'Investigacions Mèdiques, IMIM‐Parc de Salut Mar Barcelona Spain; ^4^ Reference Unit for Inherited Metabolic Disease, Hospital Universitario “12 de Octubre” Madrid Spain

**Keywords:** adult patients, Delphi consensus, follow‐up recommendations, Gaucher disease, pediatric patients, pregnancy

## Abstract

Management of Gaucher disease (GD) is challenging due to its wide genotypic and phenotypic variability and changing clinical manifestations due to effective treatment. Sixteen face‐to‐face meetings with experts were held in order to discuss daily clinical practice and identify controversies regarding the management of GD. With this information, a questionnaire with 93 recommendations for different clinical scenarios was designed, and a Delphi survey among 86 physicians with experience in GD was conducted. Consensus was reached on 73 out of the 93 items. Recommendations on follow‐up of adult and pediatric patients were in line with current guidelines, and underscored the importance of a patient‐tailored approach. For the follow‐up of stable patients receiving long‐term treatment, consensus was reached on the importance of multidisciplinary care that involves pediatricians, internal medicine, and primary care, specialized radiologists, orthopedic surgeons, and hematologists when required. Degree of pain, use of painkillers and antidepressants, and quality of life should be evaluated at every follow‐up visit or at least once per year. In general, a closer follow‐up was recommended for untreated patients or patients who underwent a treatment change (every 3 months during the first year) and during pregnancy. For pregnant patients, hemostasis and risk of hemorrhage should be assessed, but no consensus was reached for initiation of treatment in asymptomatic pregnant patients. Lastly, recommendations on how to adapt GD management during a COVID‐19 pandemic were collected. This expert consensus may help decision‐making during the management of GD in specific clinical scenarios.

**Synopsis**

Recommendations for GD management in uncertain scenarios in Spain defined through a Delphi Consensus.

## 1 INTRODUCTION

Gaucher disease (GD) (OMIM 230800, 230900, 231000) is a rare autosomal recessive disease caused by mutations in the gene encoding for glucocerebrosidase (*GBA*) (EC 3.2.1.45, OMIM 606463) that result in enzyme deficiency. Lack of glucocerebrosidase leads to the accumulation of glucosylceramide and potentially pathogenic secondary substrates in macrophages lysosomes, causing a systemic and heterogeneous disease.^1^, ^2^, ^3^, ^4^

GD presents a high phenotypic heterogeneity; thus, standard protocols for its follow‐up cannot be developed, resulting in a challenging disease to manage. GD is characterized by anemia, thrombocytopenia, hepatomegaly, splenomegaly, and bone disease; however, variable phenotypic heterogeneity ranging from intrauterine death to asymptomatic elderly people has been reported.^4^ Other clinical manifestations, such as pulmonary involvement, coagulation abnormalities, and neurologic disease have also been described.^1^, ^2^, ^4^ A range of comorbidities including hematological malignancies (multiple myeloma and non‐myeloma), neurological, metabolic, immunological, and gastrointestinal disorders have been reported in patients with GD.^5^ Traditionally, GD is divided into three subtypes according to the extent of neurological involvement: type 1 (GD1), which is the non‐neuronopathic variant and accounts for more than 90% of all GD cases; type 2 (GD2), or acute neuronopathic variant; and type 3 (GD3), or chronic neuronopathic variant.^3^, ^6^

Effective treatments for GD exist and include enzyme replacement therapy (ERT) and substrate reduction therapy (SRT).^7^, ^8^ Both treatments have been shown to be effective in improving bone, hematological, and visceral disease parameters.^9^

Due to the wide variability on clinical presentations, personalized therapeutic goals and monitoring should be established. Recommendations for GD diagnosis, management, and follow‐up have been published for adults^10^, ^11^, ^12^, ^13^, ^14^, ^15^, ^16^, ^17^ and children,^18^, ^19^, ^20^ and include treatment of current manifestations, prevention of primary and secondary manifestations, and surveillance.^14^, ^21^ In Spain, GD (mostly GD1) affects around 342 patients.^22^ Overall, Spanish patients with GD1 have good control over hematological and visceral parameters; however, a need to improve monitoring and treatment of bone disease has been reported.^22^

The aim of the present study was to find uncertain scenarios on the clinical practice of GD and use a Delphi survey to define consensus on recommendations for these specific clinical scenarios.

## 2 MATERIAL AND METHODS

**FIGURE 1:**
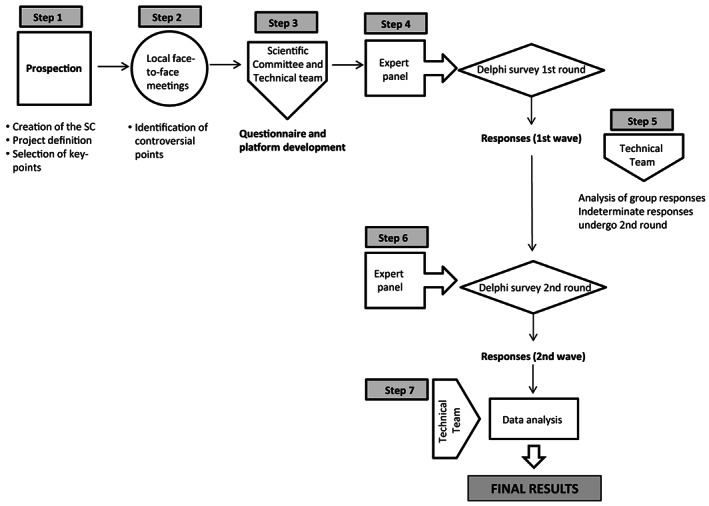
Flowchart of the study

We used a modified Delphi method^23^ to obtain a consensus among a panel of experts on management of patients with GD in Spain. This Delphi approach was carried out in seven successive phases: prospection (creation of the scientific committee [SC], project definition, selection of key‐points), local face‐to‐face meetings, design of the Delphi questionnaire, panelists' recruitment, Delphi survey administration (two rounds), data collection, and statistical data analysis (Figure 1).

A total of 16 face‐to‐face meetings with four experts and a moderator consisting of talks and participative workshops were held from October 19, 2020 to January 21, 2021. In these meetings, clinical practice with GD patients was discussed and controversial points about management identified. Based on the results from the meetings, relevant literature, and clinical expertise, the SC designed the questionnaire for the Delphi survey. The selected areas to be included in the survey were selected after the initial meetings according to the opinions of the participating specialists. The SC included three experts in management of GD (M.A‐C, M.M., and P.G.).

The final questionnaire included 76 items on the management of adults with GD and 17 items on the management of children with GD, and covered five main clinical scenarios: adult patients without treatment or with a treatment change (in type or dose) (11 items); stable patients with long‐term treatment (39 items); pregnancy, breastfeeding, and postmenopause (19 items); pediatric patients (17 items); and COVID‐19 pandemic (7 items).

Panelists with expertise on GD management from different regions of Spain were included in the study. The list of participating panelists is shown in Table S1. The Delphi questionnaire was administered through an online platform and was held to two rounds of voting, between March 15, 2021 (first wave) and April 26, 2021 (second wave), and closing date: June 7, 2021.

Descriptive analysis of the results was performed for the Likert‐type questionnaire's items with a 9‐position scale: 1–3 (disagreement), 4–6 (neither disagreement nor agreement), and 7–9 (agreement). Median score, average score, interquartile range, and percentage of panelists whose score was outside the median were calculated for each item. When less than a third of panelists (33.3%) ranked a statement outside of the median value, an item was considered to have reached “consensus” (66.7%). When a third or more panelists ranked a statement outside of the median value, the level of consensus for that item was considered as “undetermined.” When a third or more panelists ranked a statement as “agreement” and a third or more panelists as “disagreement,” the level of consensus for that item was considered as “discrepancy.” All items with an “undetermined” level of consensus or with an interquartile range ≥4 on the first round were sent for a second round of vote. Final results of Delphi survey were further evaluated and discussed by the SC.

The project was scientifically endorsed by SEMI (the Spanish Society of Internal Medicine) and SEHH (the Spanish Society of Hematology and Hemotherapy).

## 3 RESULTS

A total of 86 physicians with expertise in GD care from all over Spain were included in the panel and completed the two rounds of the Delphi survey. The items regarding the follow‐up of GD in pediatric population were only responded by professionals involved in pediatric management. Demographic characteristics and clinical medical specialties of participants can be found in Table S2. At the end of the Delphi process, consensus was reached on 73 out of 93 items (69 on “agreement” and 4 on “disagreement”). Of the 20 remaining items where panelists did not reach consensus, 17 remained with an “undetermined” level of consensus, and 3 raised a significant difference of opinion among panelists and were labeled as “discrepancies.”

### 3.1 Follow‐up of untreated GD patients and GD patients with a change in dose or type of treatment

In the follow‐up of newly‐diagnosed untreated GD patients and in GD patients with a treatment change (in type or dose), it was recommended that: (i) During the first year, clinical analytical follow‐up should be performed every 3 months, including standard biochemical analysis, clinical visit for physical examination, and a complete blood count with differential and platelets count. (ii) Follow‐up by imaging (visceral volume) every 6 months. (iii) Follow‐up of bone involvement (magnetic resonance imaging [MRI] and dual‐energy X‐ray absorptiometry [DEXA]) every 12 months. (iv) Biomarkers every 6 months.

Overall, 73.9% of experts considered that follow‐up should be different between untreated and treated patients. In untreated patients, tailored monitoring of bone involvement, imaging tests (abdominal ultrasound [US], MRI, densitometry), and biomarkers were recommended. In patients who recently started treatment, both clinical and analytical follow‐up was recommended every 3 months (77.3% of panelists) during the first year. Clinical follow‐up of stable patients every 6 months (69.3% of panelists) was recommended. Consensus on longer times for clinical analytical follow‐up (every 6 months) during the first year, and clinical follow‐up (every 12 months) in stable patients remained undetermined. According to the experts, follow‐up of untreated patients and of patients with a treatment change should include quality of life (QoL) survey, pain scale and interrogation on painkillers, anti‐inflammatory and antidepressant drugs use at each visit. In addition, panelists agreed that all patients should be asked about family history of Parkinson's disease (Table 1).

**TABLE 1:** Follow‐up of untreated GD patients and GD patients with a treatment change (in type or dose)

Item	Median (1: disagree to 9: agree)	% consensus	Result
Follow‐up of treated and untreated patients should be different.	8	73.9	Agreement
During the first year, clinical analytical follow‐up of stable patients should be performed every 3 months.	8	77.3	Agreement
During the first year, clinical analytical follow‐up of stable patients should be performed every 6 months.	8	62.1	Undetermined^a^
In untreated patients, tailored monitoring of bone involvement and imaging tests (abdominal US, MRI, densitometry and biomarkers) is recommended.	8	70.4	Agreement
Clinical follow‐up of stable patients should be performed every 6 months.	8	69.3	Agreement
Clinical follow‐up of stable patients should be performed every 12 months.	3	62.1	Undetermined^a^
In untreated patients or in patients with a treatment change (in type or dose), a quality of life survey should be included at every follow‐up visit.	7	75.9	Agreement^a^
In untreated patients or in patients with a treatment change (in type or dose), a pain scale evaluation should be included at every follow‐up visit.	8	83.0	Agreement
Untreated patients and patients with a treatment change (in type or dose) should be asked about use of painkillers and/or anti‐inflammatory drugs at every follow‐up visit.	9	94.4	Agreement
Untreated patients and patients with a treatment change (in type or dose) should be asked about antidepressant drugs use at every follow‐up visit.	8	73.9	Agreement
Untreated patients and patients with a treatment change (in type or dose) should be asked about family history of Parkinson's disease.	8	83.0	Agreement

Abbreviations: GD, Gaucher disease; MRI, magnetic resonance imaging; US, ultrasound.

a Items that underwent a second round of vote.

### 3.2 Follow‐up of stable GD patients with long‐term treatment

#### 3.2.1 Monitoring of hematological parameters

In the follow‐up of stable GD patients with long‐term treatment who achieve therapeutic goals, it was recommended that every 6 or 12 months should be performed: (i) Hemogram with differential leukocyte count and platelet count. (ii) Basic biochemistry including liver enzymes, lipid profile, and electrophoretic pattern of serum proteins. (iii) Determination of biomarkers (quitotriosidase, CCL18/PARC, LysoGb1). (iv) Determination of vitamin D levels.

Regarding monitoring of hematological parameters in stable GD patients on long‐term treatment who develop persistent anemia, panelists agreed that it should include determination of reticulocyte count, serum levels of vitamin B12, folic acid, iron and ferritin, and haptoglobin (Table 2).

**TABLE 2:** Follow‐up of stable GD patients with long‐term treatment: monitoring of hematological parameters in patients with persistent anemia and persistent thrombocytopenia, and value of bone marrow aspirate/biopsy

Item	Median (1: disagree to 9: agree)	% consensus	Result
**Patients with persistent anemia**			
Monitoring of blood parameters in patients with persistent anemia should include reticulocyte count.	9	86.4	Agreement
Monitoring of blood parameters in patients with persistent anemia should include determination of vitamin B12 serum levels.	9	89.8	Agreement
Monitoring of blood parameters in patients with persistent anemia should include determination of folic acid.	9	88.6	Agreement
Monitoring of blood parameters in patients with persistent anemia should include determination of serum iron levels and ferritin.	9	96.6	Agreement
Monitoring of blood parameters in patients with persistent anemia should include determination of serum haptoglobin.	8	72.4	Agreement^a^
ESR is indicated as a surrogate biomarker in GD.	5	52.9	Undetermined^a^
**Patients with persistent thrombocytopenia**			
Monitoring of blood parameters in patients with persistent thrombocytopenia should include assessment of hemostasis: PT, aPTT, TT, and fibrinogen.	8	81.8	Agreement
Monitoring of blood parameters in patients with persistent thrombocytopenia should include quantification of immunoglobulins and autoantibodies.	8	73.9	Agreement
Monitoring of blood parameters in patients with persistent thrombocytopenia should include serology for CMV, EBV, parvovirus B19, herpes simplex 6, HIV, hepatitis B, and hepatitis C.	8	69.0	Agreement^a^
Monitoring of blood parameters in patients with persistent thrombocytopenia should include the study of lymphocyte populations.	5	65.5	Undetermined^a^
When access to disease‐specific biomarkers is not available, serum ferritin levels can be used as guidance.	7	68.9	Agreement^a^
When access to disease‐specific biomarkers is not available, ACE levels can be used as guidance.	7	55.2	Undetermined^a^
**Value of bone marrow aspirate/biopsy**			
In GD follow‐up, bone marrow biopsy is only considered in cases of suspected neoplasia or metastasis.	8	86.4	Agreement
In case of suspected hematological malignancy, bone marrow aspirate is better than bone marrow biopsy.	5	52.9	Undetermined^a^

Abbreviations: ACE, angiotensin‐converting enzyme; aPTT, activated partial thromboplastin time; CMV, cytomegalovirus; EBV, Epstein Barr virus; ESR, erythrocyte sedimentation rate; GD, Gaucher disease; HIV, human immunodeficiency virus; PT, prothrombin time; TT, thrombin time.

a Items that underwent a second round of vote.

For stable patients on long‐term treatment who develop persistent thrombocytopenia, comprehensive study was recommended. Panelists agreed that monitoring of blood parameters should include assessment of hemostasis, quantification of immunoglobulins and autoantibodies and a serology for cytomegalovirus, Epstein Barr virus, parvovirus B19, herpes simplex 6, human immunodeficiency virus, and hepatitis B and C (Table 2). Nevertheless, they did not reach consensus on whether assessment of lymphocyte populations should be included.

Panelists agreed that serum ferritin determination can be used as guidance when access to disease‐specific biomarkers is not available, but no consensus on using angiotensin‐converting enzyme (ACE) levels as guidance was obtained. Similarly, in GD patients with persistent anemia, no consensus was reached on whether erythrocyte sedimentation rate (ESR) is indicated as a surrogate biomarker for GD (Table 2).

Finally, panelists agreed on the value of bone marrow aspirates in cases of suspected neoplasia, but not on whether these are better than bone marrow biopsies in case of suspected hematological malignancy, with most comments favoring tailored responses and referral to hematologist (Table 2).

#### 3.2.2 Monitoring of visceral parameters

In the follow‐up of stable GD patients with long‐term treatment who achieve therapeutic goals, it was recommended that every 6 or 12 months should be performed: (i) Clinical evaluation of stable GD patients. (ii) Gold standard for visceral volume determination is MRI, but if it is not available, US can be used instead. (iii) In the follow‐up of stable GD patients carrying D409H mutation, an echocardiogram should be performed every 2 years; and for other cases, at the specialist in charge of the GD patient, with special attention to splenectomized patients, because the risk of developing pulmonary hypertension is high.

For splenomegaly and hepatomegaly follow‐up in stable patients, experts recommended annual US scan to delay the computed tomography (CT)/MRI scan to 24–36 months. Furthermore, for visceromegaly follow‐up, the use of MRI was recommended, but no consensus was reached on the use of US scan alone (Table 3). Finally, an echocardiogram every 2 years was recommended.

**TABLE 3:** GD follow‐up of stable patients with long‐term treatment: monitoring of visceral parameters and bone disease

Item	Median (1: disagree to 9: agree)	% consensus	Result
**Monitoring of visceral parameters**			
For visceromegaly follow‐up, an US exam is enough.	7	51.7	Undetermined^a^
For visceromegaly follow‐up, using MRI is recommended.	8	70.5	Agreement
For splenomegaly and hepatomegaly follow‐up, an annual US exam is recommended in order to delay the CT/MRI scan to 24–36 months.	8	69.3	Agreement
In the follow‐up of stable patients, an echocardiogram every 2 years is recommended.	8	76.1	Agreement
**Monitoring of bone disease**			
Conventional radiograph is useful for routine monitoring.	3	71.6	Disagreement
Conventional radiograph is only useful in the follow‐up if the patient shows clinical signs suggestive of bone manifestations.	7	70.1	Agreement^a^
In the follow‐up of patients with bone pain or active bone disease, an MRI should be performed every 6 months.	7	55.2	Undetermined^a^
In the follow‐up of patients with bone pain or active bone disease, an MRI should be performed every 12 months.	8	71.3	Agreement^a^
Bone symptoms should be assessed by densitometry and MRI (lumbar, femoral, hip, and symptomatic areas).	8	93.2	Agreement
In the follow‐up of patients with bone pain, CT can be a suitable exam if MRI is not available.	8	75.0	Agreement
A radiologist trained in GD pathology is recommended for both MRI and radiograph interpretation.	9	100	Agreement
The use of pain questionnaires is recommended to monitor bone involvement.	9	93.3	Agreement
The amount of painkillers taken between visits should be considered to assess the degree of pain.	9	95.5	Agreement
In the follow‐up of patients with chronic bone pain, determination of blood parameters of inflammation (ferritin and/or ESR and/or CRP) is advisable.	8	74.2	Agreement
In the follow‐up of patients with bone pain, a differential diagnosis with neuropathic pain is recommended.	8	87.6	Agreement
Follow‐up of patients with bone symptoms should be the same for patients with and without prostheses.	5	23.0	Discrepancy^a^
In the follow‐up of patients with bone symptoms and prostheses, CT is recommended.	7	57.5	Undetermined^a^
Follow‐up of patients with joint prostheses should include an orthopedic surgery specialist, who may be part of the team.	9	96.6	Agreement
For patients with joint prostheses suffering pain in the prosthetic joint area or presenting signs of infection or loss of function, a scintigraphy and a visit to the orthopedic surgery specialist is recommended.	9	95.5	Agreement
For the assessment of pain and functional status, comparing the need for painkillers between visits is recommended.	8	95.5	Agreement
The SF‐36 questionnaire is useful for routine follow‐up of the GD patient.	7	70.8	Agreement
The SF‐36 questionnaire is recommended for monitoring functional status and pain.	8	77.0	Agreement^a^
The SF‐36 questionnaire is only useful in the follow‐up of the GD patients included in clinical trials.	3	56.3	Undetermined^a^
The SF‐36 questionnaire can be given to the patient for completion at home and can be collected during the next doctor visit, at least once a year.	8	78.7	Agreement
For the assessment of functional status and pain, the use of VAS is recommended.	7	70.8	Agreement

Abbreviations: CRP, C‐reactive protein; CT, computed tomography; ESR, erythrocyte sedimentation rate; GD, Gaucher disease; MRI, magnetic resonance imaging; SF‐36, Short Form Health Survey; US, ultrasound; VAS, visual analog scale.

a Items that underwent a second round of vote.

#### 3.2.3 Monitoring of bone disease

In the follow‐up of stable GD patients with long‐term treatment who achieve therapeutic goals, it was recommended that every 2 years: (i) Follow‐up of bone disease in stable GD patients should include a densitometry every 12–24 months and after two controls, if stable, it can be delayed up to every 5 years. (ii) MRI including at least lumbar spine, pelvis, femurs, and the upper third of tibias should be performed every 12 months. (iii) In case of acute pain, targeted MRI at the site of pain should be performed (CT can be a suitable exam if MRI is not available).

In line with current recommendations, panelists agreed that assessment of bone symptoms should be done by densitometry and MRI of lumbar, femoral, hip, and symptomatic areas. When MRI is not available, CT was considered a suitable test in patients with bone pain (Table 3). Regarding monitoring of bone disease, conventional radiograph was only considered useful for follow‐up of patients with clinical signs suggestive of bone disease (70.1%), and was not recommended for routine monitoring (Table 3). In the follow‐up of the patient with bone pain or active bone disease, panelists agreed that an MRI should be performed every 12 months, but no consensus on shorter periods (6 months) was reached. All panelists agreed that a trained radiologist is recommended for both MRI and conventional radiography interpretation (Table 3).

The use of pain questionnaires was recommended to monitor bone involvement, and panelists agreed that differential diagnosis with neuropathic pain should be performed in patients presenting bone pain. Panelists agreed that inflammatory blood parameters (ferritin and/or ESR and/or CRP) should be assessed in patient with chronic bone pain to assess inflammation. To assess the degree of pain, a comparison of the need for painkillers between visits was recommended, and the amount of painkillers taken between visits should be considered. Functional status of these patients should be monitored with the Short Form Health Survey (SF‐36) questionnaire and visual analog scale (VAS) (Table 3).

Panelists differed on their opinion on whether the follow‐up of patients with bone symptoms and prostheses should be the same as for patients without prostheses, and consensus remained undetermined for recommendation of CT use in these patients (Table 3). For the follow‐up of patients with joint prostheses, consulting an orthopedic surgery specialist was recommended (Table 3).

### 3.3 Follow‐up of GD during pregnancy, breastfeeding, and in postmenopause

In the follow‐up of GD during pregnancy, it was recommended that: (i) In GD patients planning a pregnancy, it was recommended to withdraw bisphosphonates and eliglustat/miglustat 2–3 months in advance. (ii) The couple has to undergo genetic testing to rule out GBA variant carrier status and genetic counseling. (iii) Pregnant woman should be informed that all her offspring will be obligate carriers. (iv) Information and close collaboration with the gynecology specialist during pregnancy and delivery was recommended. (v) Use of ERT during pregnancy is safe and it must not be stopped.

Panelists agreed that pregnancy follow‐up in GD patients should be different than follow‐up for other pregnant women. In the follow‐up of pregnant GD patients, an assessment of the general GD status in early pregnancy was recommended. Close collaboration with the gynecologist throughout gestation and delivery and closer follow‐up (monthly). In addition, closer monitoring of GD, including determination of chitotriosidase and lysoGb1 (every 3 months), monitoring of bone manifestations, and close monitoring of pregnancy was also recommended (Table 4).

**TABLE 4:** Follow‐up of GD during pregnancy, breastfeeding and postmenopause

Item	Median (1: disagree to 9: agree)	% consensus	Result
**Follow‐up during pregnancy and breastfeeding**			
In the follow‐up of pregnant patients, an assessment of the general GD status in early pregnancy is recommended.	9	94.4	Agreement
In the follow‐up of pregnant patients with GD, the same follow‐up as for other pregnant women is recommended.	3	70.8	Disagreement
A close follow‐up (once a month) and close collaboration with the gynecologist is recommended throughout gestation and delivery.	7	71.9	Agreement
In pregnant patients, closer monitoring of GD is recommended (every 3 months).	8	85.4	Agreement
In the follow‐up of pregnant patients, monitoring of vitamin B12, calcium, and folic acid levels in the first term of pregnancy is recommended.	9	97.8	Agreement
In the follow‐up of pregnant patients, the determination of chitotriosidase and lysoGb1 should be performed every 3 months.	7	73.6	Agreement^a^
In the follow‐up of pregnant patients, closer monitoring of pregnancy is recommended.	9	87.6	Agreement
In the follow‐up of pregnant patients, an assessment of the general situation of the disease should be carried out in the third term of pregnancy, including assessment of hemostasis and hemorrhagic risk.	9	95.5	Agreement
In the follow‐up of pregnant patients, vaginal delivery is recommended over cesarean section.	8	74.2	Agreement
If the pregnant patient is not on ERT, starting treatment at 60 IU/kg should be considered to avoid complications.	7	50.6	Undetermined^a^
In pregnant patients, bone manifestations should be monitored.	8	75.3	Agreement
In pregnant patients, breastfeeding should be recommended in the same way as to other pregnant women.	8	78.7	Agreement
Close collaboration with the gynecologist throughout gestation and delivery is recommended.	9	97.8	Agreement
In the breastfeeding period, breastfeeding should be only recommended for the first 6 months.	5	44.8	Undetermined^a^
In the breastfeeding period, presence of osteoporosis should be considered for breastfeeding recommendation.	8	81.6	Agreement^a^
In the breastfeeding period, continuation with ERT is recommended.	8	92.1	Agreement
**Follow‐up of postmenopausal women**			
Follow‐up of GD in postmenopausal women should include the primary care team.	8	88.8	Agreement
Follow‐up of postmenopausal women should be different than the follow‐up of other GD patients.	7	67.8	Undetermined^a^
For the follow‐up of postmenopausal women, a gynecologist should be included in the team.	7	54.0	Undetermined^a^

Abbreviations: ERT, enzyme replacement therapy; GD, Gaucher disease.

a Items that underwent a second round of vote.

In the third trimester of pregnancy, an assessment of the general situation of the disease should be carried out, including an assessment of hemostasis and hemorrhagic risk (Table 4). Finally, panelists favored vaginal delivery over cesarean section. Panelists did not reach consensus on whether a pregnant patient who is not on ERT should be always prescribed this treatment to avoid complications (Table 4).

Panelists agreed that breastfeeding should be equally recommended to women with and without GD, but that presence of osteoporosis should be taken into account. No consensus was reached on whether breastfeeding should last only the first 6 months in GD patients. On the other hand, continuation with ERT in the breastfeeding period was recommended (Table 4).

In the follow‐up of GD in postmenopausal women, it is mandatory and it is established: (i) To pay special attention to the assessment of bone mineral density. (ii) A balanced diet rich in calcium‐containing foods, avoid tobacco consumption, moderated alcohol consumption, and regular physical exercise is recommended. (iii) To perform a densitometry annually. (iv) In case of osteoporosis, to consider the administration of bisphosphonates or other agents that reduce the loss of bone mass. (v) It is recommended to monitor calcium levels and vitamin D3 deficiency.

Regarding postmenopause, experts agreed that follow‐up of postmenopausal women with GD should include specialists from primary care, but no consensus was reached on whether a gynecologist should also be included. In addition, panelists did not reach consensus on the need for a different follow‐up in patients with GD during menopause (Table 4).

### 3.4 Follow‐up of GD in pediatric population

In the follow‐up of pediatric GD patients, it was recommended that: (i) All patients diagnosed or showing symptoms at pediatric age will require ERT. (ii) Pay special attention to the growth profile. (iii) Perform neurological examination with special attention to eye movements, particularly in patients with genetic variants associated with GD2 and GD3. (iv) Insist on completing a family study to rule out other affected relatives. (v) For the follow‐up of blood parameters, a protein study should be included, especially immunoglobulin quantification.

In stable pediatric patients meeting therapeutic goals, follow‐up was recommended every 6 months, and not every 12 months (Table 5). Panelists agreed on recommending a follow‐up every 3 months in newly diagnosed patients, and routine neurological assessment in stable pediatric patients to rule‐out GD2 and GD3 variants. Panelists differed on their opinion on the need to perform an annual echocardiogram in these patients (Table 5).

**TABLE 5:** Follow‐up of GD in pediatric population

Item	Median (1: disagree to 9: agree)	% consensus	Result
In stable pediatric patients who meet therapeutic goals, a follow‐up every 6 months is recommended.	8	90.9	Agreement
In stable pediatric patients who meet therapeutic goals, annual follow‐up is recommended.	3	72.7	Disagreement
In pediatric patients 16 years and older, a bone marrow MRI is recommended every 12 months (unless a bone marrow crisis is suspected).	5	27.27	Discrepancy^a^
In pediatric patients 16 years and older, a bone marrow MRI is recommended every 24 months (unless a bone marrow crisis is suspected).	5	45.45	Undetermined^a^
Bone marrow MRI is recommended for children <16 years old who present clinical manifestations that require it.	9	90.9	Agreement^a^
In pediatric patients, at least the lower limbs, spine, hip, and pelvis should be included in the MRI.	9	90.9	Agreement
In pediatric patients, tibia MRI gives useful information in patients <9 years old.	8	81.8	Agreement^a^
In the follow‐up of stable pediatric patients, routine neurological assessment is recommended.	9	81.8	Agreement
In the follow‐up of stable pediatric patients, an annual echocardiogram is recommended.	6	9.1	Discrepancy^a^
Follow‐up of newly diagnosed pediatric patients is recommended every 3 months.	8	90.9	Agreement
For the follow‐up of pediatric patients, conventional radiograph is recommended.	2	72.7	Disagreement^a^
For the follow‐up of pediatric patients with bone pain, a densitometry should be performed annually.	8	72.7	Agreement
For the follow‐up of pediatric patients with bone pain, a MRI should be performed annually.	8	72.7	Agreement
In pediatric patients, the Calix Score 2012 questionnaire is useful for the assessment of functional status and pain.	6	36.4	Undetermined^a^
In pediatric patients, standard deviations rather than percentiles should be used for the assessment of functional status and pain.	7	72.7	Agreement
In pediatric patients, the PEDSQL quality of life test is useful for the assessment of functional status and pain.	8	100	Agreement
Completion of the family study to rule out other affected persons is recommended.	9	100	Agreement

Abbreviations: GD, Gaucher disease; MRI, magnetic resonance imaging; PEDSQL, Pediatric Quality of Life Inventory.

a Items that underwent a second round of vote.

Experts did not recommend conventional radiograph for pediatric follow‐up.

For monitoring of bone disease, bone MRI that includes at least lower limbs, spine, hip, and pelvis was recommended. In patients younger than 9 years old, tibia MRI was also considered useful.

Regarding the frequency of monitoring, in pediatric patients with bone pain, a densitometry and a MRI should be performed annually, if possible, considering the child's age (Table 5).

According to clinical practice, for patients younger than 16 years old who show clinical manifestations without bone symptoms, MRI of the bone marrow was recommended. However, in children 16 years and older, no consensus was reached on the frequency of bone marrow MRI: opinion of panelists was divided on whether it should be performed every 12 months, and consensus remained undetermined on performing a bone marrow every 24 months.

All panelists recommended assessing functional status and pain with the Pediatric Quality of Life Inventory (PEDSQL), but no consensus was reached on use of the Calix Score 2012 questionnaire. Lastly, all experts recommended completing family study to rule out other affected family members (Table 5).

### 3.5 COVID‐19 pandemic recommendations for Gaucher patients in Spain

Recommendations for the follow‐up of GD patients in Spain during the COVID‐19 pandemic included: (i) Prioritize safety but maintaining the follow‐up in the defined guidelines and preventing patients from losing treatment continuity. (ii) If face‐to‐face visits are not possible, telephone or telematic contact should be considered until face‐to‐face visits can be recovered. (iii) Ensure that splenectomized patients have completed the vaccination protocol against respiratory transmitted germs (pneumococcus, meningococcus, haemophilus, and influenza).

At the time of the study, COVID‐19 pandemic started and it became an important discussion topic among physicians treating GD patients. Therefore, statements over GD management during COVID‐19 pandemic were included in the Delphi questionnaire. Panelists agreed that in times of pandemic, switching from enzyme infusion to oral treatment as well as home therapy (if available) should be considered. Experts recommended COVID‐19 vaccination to the whole GD population, and specially to splenectomized population, as well as vaccination against other respiratory‐transmitted germs including influenza, pneumococcus, meningococcus, and haemophilus. The splenectomized patient should be evaluated and followed more rigorously due to the high incidence of irreversible bone complications and the possibility of developing pulmonary hypertension and venous thrombosis (Table S3).

## 4 DISCUSSION

GD prognosis has considerably improved since the first ERT was approved for the treatment of GD three decades ago; however, management of GD patients is still a challenge to physicians due to its complex and variable phenotypes.^4^, ^8^ In this study are presented consensus on recommendations for GD management in different clinical scenarios from a Delphi panel of physicians experienced on GD care in Spain.

GD diagnosis is elusive: signs and symptoms may mimic hematologic conditions and a significant amount of patients experience diagnostic delays of ≥5 years,^24^ which imply unnecessary testing and psychological stress, and prevent timely access to treatment.^24^, ^25^ ERT and SRT do not prevent neurological manifestations, and treatment for patients with GD2 is usually supportive.^8^ For patients with GD1 and GD3, early diagnosis and access to therapy is crucial to prevent irreversible complications such as splenectomy and reduce risk of bone crises and fractures.^7^, ^8^, ^26^ In this line, panelists recommended a closer follow‐up (every 3 months) during the first year after diagnosis in adult and pediatric patients. Similarly, panelists agreed that a close follow‐up is recommended for untreated patients, stable patients, and for patients with changes in the dose or type of treatment.

For adequate follow‐up, reliable measures to monitor disease progression are needed. Currently used markers to monitor GD disease progression are chitotriosidase and lysoGb1.^7^, ^8^ Panelists agreed that, when access to disease‐specific biomarkers is not available, serum ferritin but not ACE can be used as guidance. Nevertheless, most comments of panelists and the SC underscored the importance of the use of up‐to‐date and specific biomarkers in the management of GD. Similarly, imaging techniques such as bone MRI and densitometry are widely used and preferred for the monitoring of bone disease, and abdominal MRI for the monitoring of visceromegaly. In this sense, panelists favored MRI over US for visceromegaly follow‐up. While in adult population an echocardiogram every 2 years was recommended, panelists differed on the need to perform an annual echocardiogram in pediatric population. This may be due to the fact that there are no pediatric guidelines recommending when and how perform an echocardiogram.

Panelists did not recommended the use of CT scan and conventional radiograph for the follow‐up of bone disease, because CT does not provide information on bone infiltration and radiograph is not sensitive enough.^12^, ^17^ In clinical practice, accurate and confident identification of bone infiltration by scanning may not be so clear. Furthermore, bone MRI can be challenging in pediatric population because GD‐related infiltration may be confused with normal marrow conversion or heterogeneous residual red marrow. Even so, in current practice MRI is routinely performed because it is useful for symptomatic patients, albeit keeping in mind potential associated risks of confusion. The use and frequency of bone MRI for bone disease monitoring on pediatric patients 16 years and older was more controversial, and no period could be agreed.

Assessment of inflammatory markers was also recommended for patients with chronic bone pain.

Visceromegaly, anemia, thrombocytopenia, and bone disease are common manifestations in GD patients. Recommendations made by the panel of experts for the follow‐up and management of these manifestations for adult and pediatric patients followed current guidelines.^9^, ^10^, ^11^, ^12^, ^13^, ^14^, ^15^, ^16^, ^17^, ^18^, ^19^ For stable patients with persistent anemia or thrombocytopenia, panelists also recommended to rule‐out other causes not related to GD, such as *Helicobacter pylori* infection or autoimmune thrombocytopenia. Persistent anemia in patients on ERT is usually associated to concomitant pathologies and, in agreement with that, panelists recommended a series of tests to assess iron deficiency or viral infections.^14^, ^26^ Similarly, experts recommended close monitoring of hematological parameters that could indicate malignancy, but did not reach an agreement on specific use of bone marrow aspirate and bone marrow biopsy. Most of the panelists and the SC commented that referral to hematologist was indicated in these cases, emphasizing the importance of multidisciplinary care. Since GD patients have an increased risk of developing blood malignancies (3.5–12.7 than controls) and solid tumors such as renal cell carcinoma and hepatocellular carcinoma, early detection of malignancies is a desired therapeutic goal.^1^, ^15^, ^27^ In addition, it is recommended to perform a serum protein study through a proteinogram on an annual basis, given the high incidence of monoclonal gammopathies in these patients and the associated risk of progression to multiple myeloma. Besides, annual neurological assessment (including eye movement disorders and peripheral neuropathy) is recommended for early detecting of Parkinson disease, because it is one of the most frequent complications in GD patients.^27^, ^28^

Bone disease is one of the most unpredictable manifestations of GD, and adequate follow‐up is needed to optimize treatment, reduce risk of bone crises, and manage bone pain. Importantly, a recent study reported that follow‐up of bone disease was not adequate in up to a third of GD patients in Spain.^22^ In contrast, recommendations on bone disease management made by panelists in our study followed published recommendations.^12^, ^17^ Experts recommended involvement of radiologists with experience in GD and orthopedic surgeons in specific cases, such as in patients with prostheses. CT is a useful technique for assessing prosthetic complications rather than bone disease evaluation. Moreover, panelists recommended routine assessment of pain and use of painkillers, QoL, and functional status as described also for stable patients. In this sense, recent studies highlight the importance of using patient reported outcomes in the management of GD, as they give information of the impact of disease and effect of management on the patient.^8^, ^29^ Finally, it would be recommended to include Centers of expertise in the management of Gaucher disease as reference centers in the follow‐up of GD patients. Moreover, network with different healthcare professionals working in different areas is also recommended, in order to standardize the follow‐up of GD patients.

Some studies have assessed the effect of GD on pregnancy and the pregnant mother and reported that most of pregnancies are uncomplicated.^30^, ^31^, ^32^ Panelists agreed that close monitoring of both pregnancy and GD, particularly bone disease and hematologic risk assessment, were recommended. A multidisciplinary care with the Gaucher specialist, obstetrician, and anesthesiologist is recommended. However, no consensus was reached on a preventive start of treatment in asymptomatic pregnant patients. In this sense, current guidelines do not recommend a different approach to the start of ERT during pregnancy.^30^, ^31^, ^32^ In breastfeeding GD patients, SRT is contraindicated.^33^, ^34^ Besides, no consensus was reached on whether breastfeeding should last only the first 6 months, although the SC agreed that breastfeeding could continue in case of patients with stable disease.

Finally, it has to be noted that at the time of the study, COVID‐19 pandemic was an important discussion topic among physicians treating GD patients. Therefore, a section regarding GD patients during pandemics was included. Nevertheless, with up‐to‐date evidences regarding SARS‐CoV‐2 vaccination, some recommendations may be out of date.

Current guidelines/recommendations on GD, including the expert recommendations agreed in this study, should always be taken as guidance. An individualized approach to GD management is indispensable and is linked to the myriad of clinical manifestations and scenarios of the disease. Advances in knowledge of GD pathophysiology, discovery of new biomarkers of disease progression, and the advent of new therapies will help on further tailoring the management of GD patients.

## 5 CONCLUSIONS

The high variability of clinical phenotypes in GD makes management of the disease difficult. Despite the efficacy of current therapies on preventing disease progression and their ability to reverse some disease manifestations, GD still causes significant morbidity and reduces patients' QoL, especially in those patients with more severe phenotypic manifestations or who started treatment at more advanced stages. This study provided general recommendations on the management of GD in different clinical scenarios that may help decision‐making in GD clinical practice.

## FUNDING INFORMATION

This project has been supported by Sanofi, which has provided funds for the logistics of the meeting and the assistance with the medical writing. The authors received no financial support for the research, authorship, and/or publication of this article.

## CONFLICT OF INTEREST

Pilar Giraldo reports personal fees for lectures, advisory boards, and research project from the following pharmaceuticals: Alexion, Amicus, Chiesi, Pfizer, Sanofi, and Takeda. The financial contributions are dedicated to research on Gaucher and other lysosomal diseases through the Spanish Foundation for the Study and Therapeutics of Gaucher and other Lysosomal Diseases (FEETEG). Marcio Andrade‐Campos reports he is an employee of AstraZeneca. Montserrat Morales reports personal fees for consultancy, support for research projects, and transport for conference attendance from the following companies: Genzyme Corporation, Alnylam, and Takeda.

## ETHICS STATEMENT

This article does not contain any studies with human or animal subjects performed by any of the authors.

## Supporting information


**Table S1.** List of participating panelists of the SEGA Group
**Table S2.** Characteristics of participant experts
**Table S3.** COVID‐19 pandemic recommendations for GD patients in Spain

## Data Availability

The data that support the findings of this study are available on request from the corresponding author. The data are not publicly available due to privacy or ethical restrictions.
