# Supplementary material for: Recommendations on the follow‐up of patients with Gaucher disease in Spain: Results from a Delphi survey
Source: JIMD Rep. 2022 Nov 8;64(1):90–103. doi: 10.1002/jmd2.12342 (PMC9830018; doi:10.1002/jmd2.12342)
Supplement: Supplementary file 1 — Table S1. List of participating panelists of the SEGA Group Table S2. Characteristics of participant experts Table S3. COVID‐19 pandemic recommendations for GD patients in Spain [file JMD2-64-90-s001.pdf]

SUPPLEMENTARY TABLES

TABLE S1 List of participating panelists of the SEGA Group

Albarracín Arraigosa	Antonio
Andrade Campos	Marcio
Arévalo Gómez	Ana
Arribas Arnaiz	Ana Isabel
Bárez García	Abelardo
Blasco Alonso	Javier
Blázquez Goñi	Cristina
Bolaños Calderón	Estefanía
Cáceres Sansaloni	M.ª Desamparados
Calderón Sandubete	Enrique
Calle Gordo	María Victoria
Camprodon Gómez	María
Carrillo Linares	Juan Luis
Castro Guardiola	Antoni
Ceberio Hualde	Leticia
Correcher Medina	Patricia
Cotos Canca	Rafael
Cuenca Gómez	José Ángel
de Castro López	María José
de las Heras Montero	Javier
del Toro Riera	Mireia
Díaz Sánchez	Matías
Fernández Canal	María Cristina
Fernández Cofrades	Eva
Fernández de la Puebla	Rafael
Fernández Fontecha	Elena
Fernández Galán	María Ángeles
Fernández Martin	Julián
Gainza González	Eukene
García Frade	Luis Javier
García Hernández	M.ª Carmen
Gil Campos	Mercedes
Gil Sánchez	Ricardo
Giraldo Castellano	Pilar
Godoy Molías	Ana Cristina
Gómez Núñez	Marta
Gutiérrez Macías	Alfonso
Hermosín Ramos	Lourdes
Hernández Rivas	Jesús María

Ibarretxe Gerendiaga	Daiana
Iglesias Julián	Enrique
Iglesias Pérez	Ana María
Labbadia	Francesca
Lakhwani	Sunil
Madinaveitia Ochoa	Andrés
Manresa Manresa	Pablo
Marco Amigot	Javier
Martín Hernández	Elena
Martínez Revuelta	Eva
Méndez Sánchez	José Ángel
Mora Casterá	Elvira
Morado Arias	Marta
Morales Conejo	Montse
Nieto Fernández	Santiago
Noya Pereira	María soledad
Núñez Vázquez	Ramiro José
O’Callaghan Gordo	María Del Mar
Ojeda Sosa	Ana
Ordieres Ortega	Lucía
Pascual Izquierdo	Cristina
Peña Quintana	Luis
Pérez de León	José Antonio
Pérez De Pedro	Iván
Pérez García	María Luisa
Pérez Marín	Juan Carlos
Pérez Montaña	Albert
Pijierro Amador	Agustín
Rivera Gallego	Alberto José
Rivera García	Susana
Rodríguez Fernández	Alicia
Rodríguez López	Manuel Isidro
Roig Martínez	Inma
Romero Carmona	Rafael
Ruiz de Gaona	Estefanía
Sánchez Martínez	Rosario
Sánchez Sánchez	Sara
Saura Grau	Salvador
Solanich Moreno	Xavier
Suárez Álvarez	Carmen
Tormo Díaz	Mar
Vélez	Mónica
Verdu Berenguer	Alicia

Villalón	Lucia
Villarrubia Espinosa	Jesús
Visa Reñe	Nuria
Vitoria Miñana	Isidro

TABLE S2 Characteristics of participant experts

	**N = 86**
**Gender, n (%)**	
Male	38 (44.2%)
Female	48 (55.8%)
**Age, n (%)**	
30-40 years	16 (18.6%)
41-50 years	24 (27.9%)
51-60 years	27 (31.4%)
>60 years	12 (14.0%)
ND	7 (8.1%)
**Geographical distribution, n (%)**	
Andalusia	16 (18.6%)
Aragon	4 (4.7%)
Asturias	2 (2.3%)
Basque Country	5 (5.8%)
Canary Islands	6 (7.0%)
Castile - La Mancha	1 (1.15%)
Castile and León	7 (8.1%)
Catalonia	11 (12.8%)
Community of Madrid	9 (10.5%)
Extremadura	2 (2.3%)
Galicia	9 (10.5%)
La Rioja	1 (1.15%)
Region of Murcia	3 (3.5%)
Valencian Community	10 (11.6%)
**Medical specialty**	
Hematology hemotherapy	42 (48.8%)
Internal medicine	32 (37.2%)
Pediatrics and specific areas	11 (12.8%)
Medical oncology	1 (1.2%)

ND: not determined

TABLE S3 COVID-19 pandemic recommendations for GD patients in Spain

**Item**	**Median** (1: disagree - 9: agree)	**% Consensus**	**Result**
During a pandemic we must consider switching enzyme infusion to oral treatment.	7	67.8%	Agreement*
During a pandemic we should consider spacing out the enzyme treatment regimen (from 14 days to 28 days) while maintaining the total cumulative dose.	7	64.4%	Undetermined*
During a pandemic we must consider home therapy in case of being available in our autonomous region.	9	98.9%	Agreement
During a pandemic we must consider sending oral treatment to the patients' home.	9	95.5%	Agreement
During a pandemic we should recommend COVID-19 vaccination for splenectomized GD patients.	9	98.9%	Agreement
During a pandemic we should recommend COVID-19 vaccination for people living with splenectomized GD patients.	8	86.5%	Agreement
During a pandemic we should recommend influenza vaccination for people living with splenectomized GD patients.	9	95.5%	Agreement

* Items that underwent a second round of vote.

GD: Gaucher disease.
